# Candida Auris Urinary Tract Infection in a Nursing Home Patient With Multicomorbidities

**DOI:** 10.7759/cureus.12322

**Published:** 2020-12-27

**Authors:** Mirian V Garcia Rivera, Jonathan J Heyl, Michael C Oh

**Affiliations:** 1 Internal Medicine, St. John's Episcopal Hospital, New York, USA; 2 Internal Medicine, Lake Erie College of Osteopathic Medicine (LECOM), Bradenton, USA

**Keywords:** candida auris

## Abstract

Candida (C.) auris is an opportunistic ascomycetous budding yeast that has been emerging as an invasive, multidrug-resistant pathogen over the past 11 years since its discovery. Candida auris infection has raised considerable attention in public health organizations due to its rising number of cases, virulence, and unique resistance to commonly used mycofungal therapy. This case follows a 64-year-old male with multiple comorbidities from the nursing home presenting with polybacterial sepsis along with a urinary tract infection growing Candida auris. Along with treatment for sepsis, the patient was placed on the Centers for Disease Control and Prevention's (CDC’s) recommended regimen of micafungin to eradicate C. auris infection and isolation precautions. Cases should be approached carefully and reported to public agencies such as the CDC and state health department.

## Introduction

Candida (C.) auris is an opportunistic ascomycetous budding yeast that has been emerging as an invasive, multidrug-resistant pathogen over the past 11 years since its discovery. It seems that there is a common underlying theme to the contraction of C. auris: high exposures to healthcare facilities, comorbid conditions, and the organisms’ intrinsic ability to evade anti-fungal regimens.

As of June 30, 2020, the total number of cases of C. auris recorded by the Centers for Disease Control and Prevention (CDC) in the United States totaled 1208. The epicenter of the spread of this deadly organism seems to take its roots in the states of New York (551 cases), New Jersey, and Illinois, which remain the major source of all confirmed cases [1].

C. auris is well-known for its resistance to common mycofungal therapy with some literature showing up to 90% of resistance to fluconazole and 73% to voriconazole [2]. Another study found that select isolates of C. auris were resistant to the common last resort drug amphotericin B at a rate of up to 35% [3]. Fortunately, C. auris is still susceptible to multiple other triazoles and the majority of echinocandins [3].

A retrospective review of isolates points to South Korea having the first known infections, dating back to 1996. Identification was done with rRNA sequencing and biochemical analyses showing a new Candida species. From 2009 to 2011, two hospitals in Delhi, India, were also found to contain patients with candidemia with high mortality rates [4].

Much research is being conducted in identifying the multidrug-resistance pattern on the microbiological scale in order to better understand its malignant spread, along with being able to identify infections with prompt treatment.

## Case presentation

The patient is an African American, 64-year-old male from a nursing home with an extensive past medical history, including diabetes mellitus, hypertension, anemia of chronic disease, chronic obstructive pulmonary disease, chronic stage four sacral decubitus ulcer complicated with osteomyelitis of the ischium, ulceration of the right ankle, coronary artery disease, prior admissions to the intensive care unit (ICU) due to septic shock, contractures, penicillin allergy, dementia, and a chronic Foley catheter who presented to the emergency department with hypotension (systolic blood pressure 68 mmHg, diastolic pressure 42 mmHg, and medium arterial pressure of 50 mmHg) and altered mental status. On initial laboratory results, the patient was found to have leukocytosis (white blood count of 15.7 x10^9^/L), urine analysis showed a moderate amount of leukocyte esterase, white blood count of 50, and a moderate amount of urine yeast. Suspected sources of infection were thought to be from the urinary tract, decubitus ulcer, and otitis media. This patient was initially treated with aggressive intravenous fluid resuscitation and empiric antibiotic treatment for sepsis, which included vancomycin and Azactam (due to penicillin allergy). Subsequently, the patient responded to initial treatment and did not require ICU admission, as his blood pressure increased to 88/60 mmHg with medium material pressure of 69 mmHg. Blood cultures drawn before the administration of antibiotics revealed gram-negative rods, Proteus mirabilis ESBL (extended-spectrum beta-lactamases) while the urine culture showed Candida auris along with vancomycin-resistant Enterococcus. Antibiotic and antifungal therapy was adjusted to the identified organisms. For bacteremia, the patient was started on meropenem and vancomycin. Polymyxin was later added to the management. The regimen for Candida auris was micafungin 150 mg daily. The Foley catheter was changed, and he had surgical debridement of his ulcerations along with wound care daily. This patient was considered high risk for Candida infection due to his indwelling Foley catheter, nursing home status, and several prior admissions, including one to the critical care unit. He was medically stabilized and discharged two weeks later.

## Discussion

Virulence factors and difficulty with the identification of C. auris have made this fungus incredibly dangerous when it infects a host, but arguably the most concerning part of C. auris, is its high resistance profile to popular antifungals. Over 90% of known strains of C. auris are resistant to fluconazole while up to 73% of strains are resistant to voriconazole [2]. Another study found that select isolates of C. auris were resistant to the common last-resort drug amphotericin B, at a rate up to 35% [3]. Fortunately, C. auris is still susceptible to multiple other triazoles and the majority of echinocandins [3].

The primary reason for the wide range of resistance is the overexpression of adenosine triphosphate (ATP)-binding cassette transporter (ABC) genes that were discovered in the C. auris genome [4]. ABC transporter genes use ATP binding and hydrolysis to provide energy for the translocation of substrates, which leads to an increased expulsion of the drugs out of its cells. C. auris has multiple other virulence factors, including lipases, mannosyl transferases, oligopeptide transporters, and the ability to develop a biofilm [4].

Lipases are a catalyst for the hydrolysis of the ester bonds of triacylglycerols, which allows fatty acids to be released. Candida species use these to destroy epidermal and epithelial tissues [5].

Basic information about the case patient, including demographic characteristics and clinical history, should be obtained. In the United States, patients with C. auris were found to have had an average of three healthcare facility encounters in the 90 days preceding their diagnosis; the majority had been admitted to a high-acuity long-term care facility (LTCF) [6]. It is important to obtain records of recent healthcare encounters, including stays at other acute care hospitals and LTCFs in order to assess for possible transmission at the other facilities. Taking a detailed travel history, especially the receipt of healthcare in countries where C. auris cases have been reported, is important. Several U.S. case-patients have had a recent history of hospitalization in countries with a large burden of C. auris, including India, Pakistan, South Africa, and Venezuela. Based on whole-genome sequencing at CDC, most isolates from U.S. patients are closely related to isolates from South Asia and South America [6].

Multiple factors, including the difficulty to identify C. auris, antibiotic resistance, and a plethora of virulence, make the mortality rate particularly high in infected individuals. One of the characteristics of C. auris that makes it so deadly is that it can evade the response of the innate immune system by disabling neutrophil extracellular traps (NETs) in comparison to Candida albicans [7].

The severity of C. auris infection is such that as an example, the first three infectious cases of C. auris in South Korea lead to fungemia, resulting in the death of two of these patients [8].

Due to its high rate of pathogenicity, it may be wise to bolster preventative measures such as public health safety and hospital management of identified cases. Public health agencies, such as the CDC and local health departments, should be notified of confirmed or suspected cases of C. auris. Patients should be placed in single rooms, with standard contact precautions, proper hand hygiene practices, along with proper sanitation of various surfaces in the room, including but not limited to bedside tables, bed rails, windowsills, glucometers, blood pressure cuffs, crash carts, and nursing carts. Recommended disinfectants include Environmental Protection Agency (EPA)-registered disinfectants with efficacy against C. diff spores [9]. There is paramount importance in the periodic swabbing of patients at the axilla, groin, and past infected sites. In addition, prior roommates and healthcare workers (HCW) with prolonged contact with the patient should also be swabbed to prevent the spread of infection to other susceptible patients.

The CDC recommends continuing to set appropriate transmission-based precautions for the entire duration of the patient’s stay in the facility. If a patient’s clinical status improves significantly, a reassessment of colonization may be considered in consultation with the relevant state or local public health department. Reassessments should not be performed during the three months after the patient’s last test result positive for C. auris [9]. In addition, the patient should not be receiving antifungal medications active against C. auris at the time of these assessments. The optimal time between the last receipt of antifungal medications and testing has not been established, but it is reasonable to wait one week. When reassessment is considered appropriate, the CDC recommends that C. auris-specific infection control precautions be discontinued only if a patient or resident has two negative colonization tests at least one week apart [9].

## Conclusions

Candida auris is a highly infectious yeast, which is attributable to its highly infectious nature, massive antibiotic resistance, and difficulty of identification. As it is resistant to most fluconazole classes, the mainstay of treatment includes using an echinocandin such as micafungin. If the infection has not been cleared in five days or is recurring, amphotericin B should be considered. Patients should be placed in single rooms with contact precautions to avoid spread. Public health agencies should also be notified as soon as possible.
